# Infants Learn What They Want to Learn: Responding to Infant Pointing Leads to Superior Learning

**DOI:** 10.1371/journal.pone.0108817

**Published:** 2014-10-07

**Authors:** Katarina Begus, Teodora Gliga, Victoria Southgate

**Affiliations:** Centre for Brain and Cognitive Development, Birkbeck College, University of London, London, United Kingdom; University of Portsmouth, United Kingdom

## Abstract

The majority of current developmental models prioritise a pedagogical approach to knowledge acquisition in infancy, in which infants play a relatively passive role as recipients of information. In view of recent evidence, demonstrating that infants use pointing to express interest and solicit information from adults, we set out to test whether giving the child the leading role in deciding what information to receive leads to better learning. Sixteen-month-olds were introduced to pairs of novel objects and, once they had pointed to an object, were shown a function for either the object they had chosen, or the object they had ignored. Ten minutes later, infants replicated the functions of chosen objects significantly more than those of un-chosen objects, despite having been equally visually attentive during demonstrations on both types of objects. These results show that offering information in response to infants’ communicative gestures leads to superior learning (Experiment 1) and that this difference in performance is due to learning being facilitated when infants’ pointing was responded to, not hindered when their pointing was ignored (Experiment 2), highlighting the importance of infants’ own active engagement in acquiring information.

## Introduction

The majority of current developmental models prioritise a pedagogical approach to knowledge acquisition in infancy. According to this approach, infants are on the receptive side of the pedagogical exchange, having evolved mechanisms enabling them to identify when and what adults intend for them to learn [1] [2]. Taking the leading role in learning, knowledgeable adults ensure efficient transmission of vast amounts of culturally relevant information. However, while infants appear to be well equipped to learn from adults’ teaching, and sometimes indiscriminately acquire information accompanied by ostensive cues [3] [4], it is increasingly clear that infants play a more active and solicitous role in their learning. For example, infants are selective in what they attend to [5] and in whose gaze they follow [6], as well as whose actions they imitate [7], all of which are likely to be important mechanisms in the process of cultural learning. Even during infancy, adult-led learning may not always be the optimum strategy, especially in situations where exploration and innovation are required [8].

Recent work suggests not only that infants are selective recipients, but that they also have means of actively expressing interest, eliciting communication and soliciting information, even prior to the emergence of explicit verbal questioning. Early pointing is one such means by which infants elicit information from adults. Studies have shown infants’ pointing gestures perform the function of provoking adults to comment on the referent [9] and they do so more efficiently than object-directed babbling [10]. Begus and Southgate [11] recently demonstrated that 16-month-old infants are motivated to point because they expect others to provide them with information about the referents of their gestures. In the latter study, the amount of pointing depended on an experimenter’s perceived competence to provide infants with information. Specifically, infants pointed significantly less towards novel objects when the experimenter had previously shown themselves to be unknowledgeable (i.e. had mislabeled common objects) than when the experimenter was either demonstrably knowledgeable, or the infant had no evidence of the experimenter’s competence. Infants were equally willing to interact with the experimenter irrespective of her competence, and thus it was concluded that infants expect their pointing to be responded to with reliable information, and use it only when they perceive their expectations can be met.

In adults, much work exists, which demonstrates a relationship between epistemic interest and learning, with desired information being more likely to be assimilated (e.g. [12]). While there has been no direct test of the existence of the same relationship early in life, there is some indirect evidence that infant learning might also be driven by interest. For example, there is a positive relationship between amount of pointing and vocabulary growth [13] and it has also been shown that it is the referents of infants’ points, which the caregiver likely names in response [9], that are most likely to enter the child’s vocabulary [14]. These data could be interpreted as suggesting that infants are more likely to learn the labels of referents about which they had expressed their interest in through pointing. In the current study, we aimed to directly test the hypothesis that infants will better assimilate information that is provided in response to their expressions of interest, than information that is provided in the absence of any expression of interest by the infant. Specifically, we asked whether 16-month-old infants would show superior learning when they were provided with information about a referent which they had expressed their interest in through pointing.

## Methods

### Ethics Statement

All participants were recruited from a database of infants whose parents had volunteered to participate in infant studies at Centre for Brain and Cognitive Development, Birkbeck College, University of London. Written informed consent was obtained from the infants’ caregiver before the experiment was conducted. The procedure was approved by the ethics committee of the Department of Psychological Sciences, Birkbeck College, University of London.

### Participants

Fifty 16-month-olds (20 female, range 15.2–16.2 months) participated in the study. Infants were randomly assigned to either Experiment 1 (*N* = 16) or one of the two conditions of Experiment 2 (*N* = 17 each). An additional 14 infants (8 from Experiment 1 and 6 from Experiment 2) were tested but excluded from analysis due to parental interference (4), fussiness (4), equipment failure (3) and absence of pointing (3). Because the aim of the study was to establish how responding to infants’ pointing affects their learning, only infants, who pointed at least twice during the experiment, were included in the final sample. This criterion did not apply to *No Choice* condition of Experiment 2.

### Experiment 1

#### Procedure

**Figure 1:**
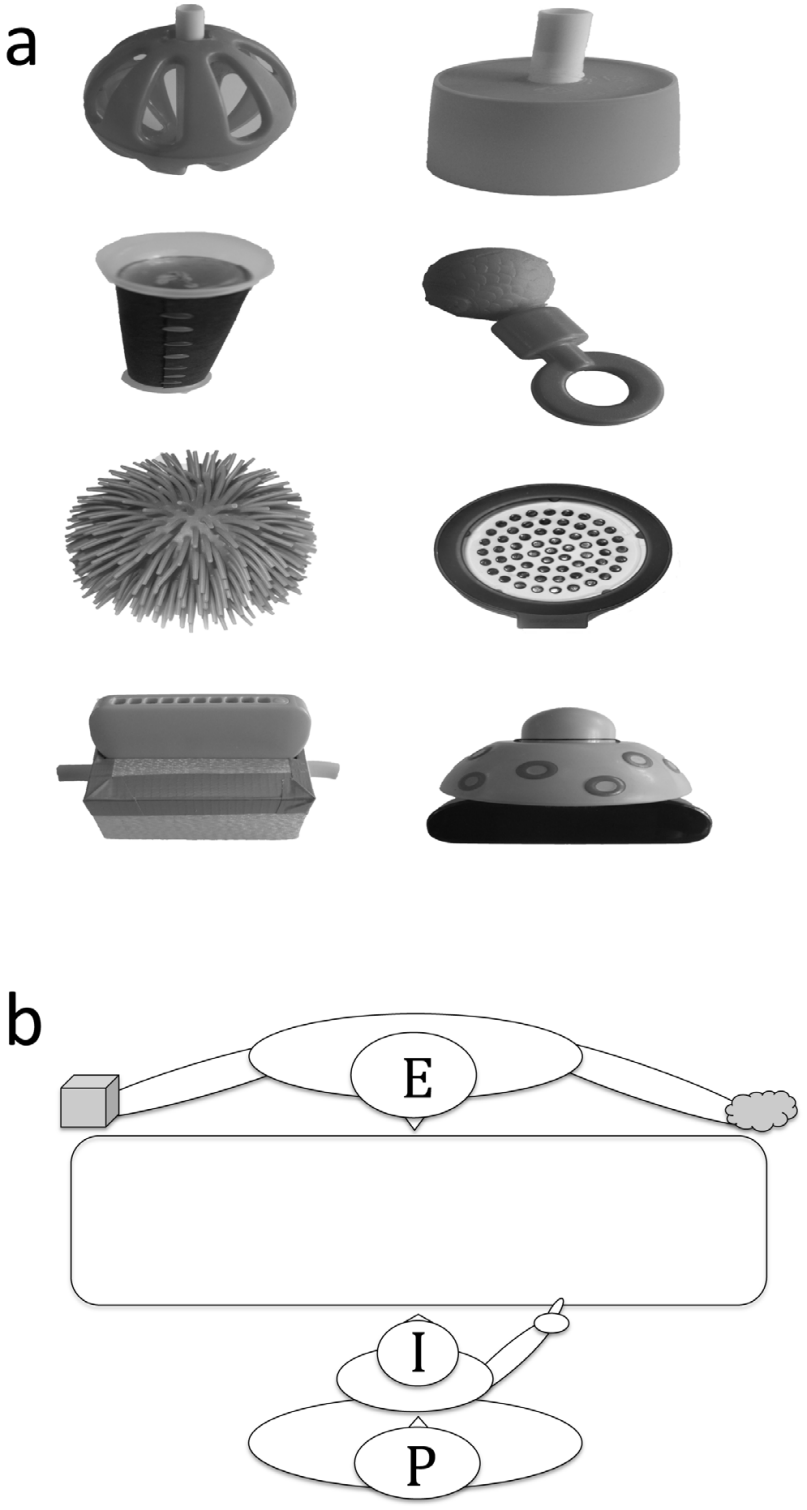
Materials and experimental set-up. (a) Photographs of the 8 novel objects used in the study. (b) Schematic representation of the experimental scene.

*Teaching phase*. Infants were presented with 4 pairs of novel objects (Fig. 1a) held at a distance until the infant pointed to one of the objects (Fig. 1b). Once infants had made their choice, the experimenter demonstrated an action either with the object the infant had chosen (*Chosen* condition, 2 trials) or with the un-chosen object (*Unchosen* condition, 2 trials), while the other object was removed from view. Each action was demonstrated a single time, with the experimenter announcing the demonstration and commenting on the action (i.e. *“Let me show you how it works! Look, I can brush my hair with it!”*). After the demonstration, the object was removed without the infant handling it.

Infants saw the same 4 actions demonstrated regardless of which object they chose. Which action was performed with which object, whether an action was performed on a chosen or un-chosen object, and whether the infant first saw a demonstration on a chosen or un-chosen object, was counterbalanced across 4 conditions (see Table S1). In order to ensure equal cognitive load, an action was demonstrated even if an infant did not point to either of the objects (which occurred on 2 trials in *Chosen* and 1 in *Unchosen* condition), however these trials were not included in further analysis.

*Testing phase.* After a 10 minute break, infants were handed each of the previously acted upon objects individually and were prompted to perform the previously demonstrated actions (i.e. *“Can you show me how it works?”*). The first 60 seconds of each trial was analysed for correctly replicated actions and other infant behaviour.

#### Data analysis

All trials, in which the infants have touched the object in the test phase, were included in the analyses. Number of trials, in which the infant refused to interact with the object for 60 seconds and were terminated earlier, did not differ between conditions (*N(Chosen)*  = 10, *N(Unchosen)*  = 11; *Mann-Whitney U*, *z* = 0.162; *p* = 0.871). Data was coded from video recordings of the testing sessions by 2 independent coders, one of whom was naïve to the experimental hypothesis. An action was scored as correctly replicated if both coders agreed the infant had performed the target action. Trials in which no target or an incorrect target action was performed were scored as incorrect. Infants had to contribute one trial of each condition (*Chosen* and *Unchosen*) to be included in the final sample (11 infants contributed 4 trials; 4 contributed 3 trials; and 1 contributed 2 trials).

#### Results

Non-parametric tests, comparing average proportions of correctly replicated actions across infants, revealed that infants replicated significantly more of the actions demonstrated on objects they had pointed to (*M* = 40.6%, a total of 13 actions across infants) than actions demonstrated on the objects they had not pointed to (*M* = 12.5%, a total of 4 actions across infants) [*Related-Samples Friedman’s Two-Way Analysis of Variance by Ranks*, *χ^2^*(*2*)* = *4.455, *p = *0.035, two-tailed].

In order to rule out that infants learned less about the *Unchosen* objects because they were attending less, we measured the time infants visually attended to the demonstrations (as a proportion of the total demonstration time), which revealed no significant difference between the conditions (time attending to demonstrations in *Chosen*: *M* = 98.9% and *Unchosen* condition: *M* = 97.0%; *t*(*55*) = 1.605; *p* = 0.114). In addition, on trials when the correct actions were not replicated, infants were equally willing to explore both *Chosen* and *Unchosen* objects in the test phase (time spent handling *Chosen*: *M* = 46.89 sec and *Unchosen* objects: *M* = 42.82 sec; *t*(*38*) = 0.898; *p* = 0.374). This suggests the number of replicated actions in *Unchosen* condition did not result from them having less visual exposure during demonstrations or less opportunities to demonstrate their knowledge during test.

Furthermore, due to the fact that the experimenter could not be blind to infants’ choice, we took some additional measures to ensure the experimenter’s behavior did not differ between conditions, which could have affected infants’ learning. We found no effect of condition on any of the measures which included the number of times, during demonstration, the experimenter a) attempted to get the infant’s attention, b) positively commented on the object/action, c) provided information; as well as d) prosody of the experimenter’s speech and e) duration of the demonstrations (see Table S2 online for statistical details).

Finally, an analysis of number of times each object was chosen confirmed that objects were distributed similarly in the *Chosen* and *Unchosen* conditions (Chi-Square comparisons for each pair: *χ^2^* = 0.6, *p* = 0.439; *χ^2^* = 1.0, *p* = 0.317; *χ^2^* = 1.923, *p* = 0.166; *χ^2^* = 0.692, *p* = 0.405). Thus differences in learning performance cannot be explained by differences in the objects themselves.

### Experiment 2

Although different rates of learning for *Chosen* and *Unchosen* objects suggest that responding to infants’ points affected their knowledge acquisition, it is unclear whether infants’ learning in Experiment 1 was *facilitated* when their pointing was responded to appropriately, or *hindered* when their pointing was ignored. To address this question, we ran Experiment 2, a between-subject control experiment, to establish how much infants learned when they did not have a choice in what they are taught (*No Choice* condition) and compared it to learning when they are given a choice of objects, and all their choices are responded to (*Chosen Only* condition). If the experimenter’s failure to respond to infants’ pointing on *Unchosen* trials of Experiment 1 is responsible for infants’ comparatively inferior learning, we should expect no difference in learning between *No Choice* and *Chosen Only* conditions, since neither involve trials in which the experimenter ignores infants’ points. However, if the provision of information in response to infants’ points on *Chosen* trials facilitates learning, we would expect infants to learn more of the object functions in the *Chosen Only* than in the *No Choice* condition.

#### Procedure

The procedure was identical to Experiment 1 except that in the *Chosen Only* condition, the experimenter always responded to infants’ points by demonstrating an action on the object the infant had chosen, and in the *No Choice* condition, instead of being presented with pairs of objects, infants were presented with single objects and subsequent demonstrations of their functions. Only infants who contributed the minimum of 2 trials were included in the final sample (29 infants contributed 4 trials, 4 contributed 3 trials, and 1 contributed 2 trials).

To ensure the conditions of Experiment 2 were equally engaging and demanding for infants, the two conditions were closely matched in all measures of the experimenter’s behaviour analysed in Experiment 1, as well as in the amount of time infants saw the objects before the demonstrations (see Table S2 online for statistical details). Number of trials, in which the infant refused to interact with the object for 60 seconds and were terminated earlier, did not differ between conditions (*N(Chosen Only)*  = 10, *N(No Choice)*  = 15; *Mann-Whitney U*, *z* = 0.635; *p* = 0.526).

#### Results

When presented with single objects and their functions (*No Choice*), infants on average correctly replicated 12.2% (total of 8 actions across all infants) of all demonstrated functions, which was significantly lower than the average of 26.0% (total of 16 actions across all infants) of correctly replicated actions in the *Chosen Only* condition (*Mann-Whitney U Test*, *z = 1.759*, *p = 0.039*, 1-tailed). The total number of replicated actions in the *Chosen Only* condition (16 across all infants) is similar to the total number or replicated actions in the *Chosen* condition of Experiment 1 (13 across all infants). Furthermore, the total number of replicated actions in the *No Choice* condition (8 across all infants) is similar to the total number of replicated actions in the *Unchosen* condition of Experiment 1 (4 across all infants).

As in Experiment 1, measures of attention during the demonstrations and time spent handling objects during the *Testing phase* revealed no differences between the two conditions of Experiment 2, ruling out visual exposure or lack of opportunity to demonstrate knowledge as an explanation of the found result (see Table S2 online for statistical details).

## Discussion

Previous work has shown a positive relationship between amount of pointing, and vocabulary growth [13] in infancy, but left unanswered the question about the mechanisms driving the relationship between gestures and learning. Our finding that 16-month-old infants replicated significantly more of the actions previously demonstrated on objects they had pointed to, than actions demonstrated on objects they had not pointed to, provides the first direct evidence that responding to infants’ gestures with appropriate information results in superior learning.

While infants’ learning was affected by whether they received information about the object they had pointed to, no other measure of behavior revealed any differences between conditions. Infants visually attended to the demonstrations equally, regardless of which object was demonstrated, and were equally willing to handle all objects. All demonstrations, regardless of condition, were equally rich in pedagogical cues (i.e. ostensive cues like mutual gaze, infant-directed speech), suggesting that the presence of ostensive cues alone was not sufficient for learning in this paradigm. Previous research has revealed that information received contingently with other infant behavior, like object-directed babbling, is learned better than information received non-contingently [15], [16] and that individual differences in parental responsiveness to infant vocalizations are reliably related to language outcomes [16]. However it remained unknown what mechanisms mediated this relationship and whether following-in on a child’s attention has a beneficial effect, or whether redirecting infants’ attention has a detrimental effect on learning. Our control Experiment 2 provides a first step towards answering this question, demonstrating that it was not the fact that the experimenter ignored infants’ pointing in the *Unchosen* trials that drove the effect in Experiment 1, but rather it is something about the situation in which infants’ pointing is appropriately responded to, that drives superior learning.

What mechanisms might mediate the relationship between pointing and learning? One possibility is that the act of making a choice is itself a factor, as suggested by findings that having a choice in the stimuli to be learned increases the learners’ perception of control and consequently enhances motivation and learning performance [18]. An alternative possibility is that what drove the superior learning in our paradigm was the same drive that motivated infants to point in the first place, namely interest. It is well established that, in adults, a positive relationship exists between interest and learning. Epistemic curiosity, as a trait, can explain individual differences in academic achievement [19], and experimental manipulations have demonstrated that degree of interest or motivation for receiving particular information determines whether that information is subsequently retained [12] [20]. For example, in Kang et al. [12], self-reported curiosity about a particular piece of information correlated with its recall 1 to 2 weeks later. We propose that a similar relationship between interest and learning may exist early in life, that interest can be expressed through pointing, and that responding to these expressions of interest plays an important role in infant learning.

Some of the earliest accounts of infant pointing suggested that the initial function of pointing is to focus infants’ own attention on interesting events [17]. Several studies since have shown that infants also use pointing to communicate their interest to others [21]. While there is still debate on what motivates infants to share their interest with others, we believe the current findings, that infants learn better when they receive information in response to their pointing, provides further evidence that one of the reasons infants express their interest is in order to obtain information about the object of interest, and that, when doing so, they may be in an optimal state for assimilating information.

However, while we believe that existing data support the conclusion that pointing is both interrogative (i.e. used to gain information, [11]) and communicative (i.e. aimed at others as information sources), it is ultimately not possible to know exactly what motivated infant pointing in this particular paradigm. Nonetheless, our conclusion, that there exists a relationship between expressions of interest and learning, even early in life, still holds even if infants were merely *requesting the object* that they were more interested in, and were not *requesting information* per se, as both kinds of pointing are motivated by interest. Regardless of what motivated infants’ pointing in this experiment, our data suggest that the extent to which infants learn information in everyday life depends, in part, on the extent to which caregivers both detect and appropriately respond to infants’ expressions of interest, such as pointing. Understanding the factors involved in learning in infancy, and the potential importance of caregiver responsiveness, may be especially relevant in situations where infants are competing for caregiver’s attention, such as in a nursery or kindergarten setting.

By presenting the first direct evidence that responding to infants’ communicative gestures affects their knowledge acquisition, we hope to open new opportunities for the study of learning in preverbal infants, with the focus on infants’ own active engagement in acquiring information.

## Supporting Information

Table S1 **Procedure.**
(DOCX)

Table S2 **Additional Analysis.**
(DOCX)
