# Supplementary material for: Infants Learn What They Want to Learn: Responding to Infant Pointing Leads to Superior Learning
Source: PLoS One. 2014 Oct 7;9(10):e108817. doi: 10.1371/journal.pone.0108817 (PMC4188542; doi:10.1371/journal.pone.0108817)
Supplement: Table S1 — Procedure. (DOCX) [file pone.0108817.s001.docx]

Table 1. *Pairs of objects and corresponding actions for each of four conditions. Actions: listen (1), pull (2), brush (3) and swipe (4).*

|  |  | **Object Pair** | **Condition 1** | **Condition 2** | **Condition 3** | **Condition 4** |
| --- | --- | --- | --- | --- | --- | --- |
| **← Time ←** | Warm-up | Familiar | Chosen | Chosen | Chosen | Chosen |
|  | Warm-up | Familiar | Chosen | Chosen | Chosen | Chosen |
|  | **Trial 1** | 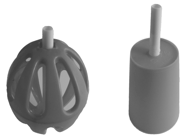 | Chosen | Unchosen | Chosen | Unchosen |
|  | Action |  | 1 | 1 | 2 | 2 |
|  | **Trial 2** | 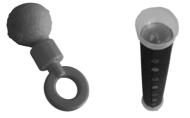 | Unchosen | Chosen | Unchosen | Chosen |
|  | Action |  | 2 | 2 | 4 | 4 |
|  | **Trial 3** | 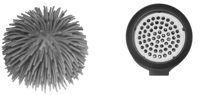 | Chosen | Unchosen | Chosen | Unchosen |
|  | Action |  | 3 | 3 | 3 | 3 |
|  | **Trial 4** | 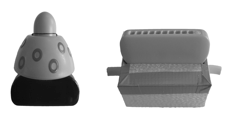 | Unchosen | Chosen | Unchosen | Chosen |
|  | Action |  | 4 | 4 | 1 | 1 |
