# Supplementary material for: Infants Learn What They Want to Learn: Responding to Infant Pointing Leads to Superior Learning
Source: PLoS One. 2014 Oct 7;9(10):e108817. doi: 10.1371/journal.pone.0108817 (PMC4188542; doi:10.1371/journal.pone.0108817)
Supplement: Table S2 — Additional Analysis. (DOCX) [file pone.0108817.s002.docx]

Table S2. Additional Analysis. *Average values for measures of infant and experimenter behaviour in Experiment 1 and 2, test statistics and significance levels of the differences between conditions (average inter-coder reliability κ = 0.838)*. ^1^

| **a** |  | **Measure** | ***M(Chosen*)** | ***M(Unchosen*)** | ***t*** | ***Sig. (p)*** |
| --- | --- | --- | --- | --- | --- | --- |
| **Experiment 1** | ***Experimenter*** | Attention Getters *(F)* | 6.28 | 6.61 | 1.047 | 0.300 |
|  |  | Positive commenting *(F)* | 1.97 | 1.82 | 0.645 | 0.521 |
|  |  | Information *(F)* | 1.76 | 1.57 | 1.392 | 0.170 |
|  |  | Prosody | 1.76 | 1.89 | 0.470 | 0.640 |
|  |  | Demo. duration *(s)* | 5.35 | 5.03 | 0.695 | 0.490 |
|  | ***Infant*** | Attention *(%)* | 98.9 | 97.0 | 1.605 | 0.114 |
|  |  | Time handling objects *(s)* | 46.89 | 42.82 | 0.899 | 0.374 |
|  |  |  | ***M(ChosenOnly*)** | ***M(NoChoice*)** | ***t*** | ***Sig. (p)*** |
| **Experiment 2** | ***Experimenter*** | Attention Getters *(F)* | 8.64 | 8.24 | 1.103 | 0.272 |
|  |  | Positive commenting *(F)* | 1.57 | 1.64 | 0.386 | 0.700 |
|  |  | Information *(F)* | 1.43 | 1.53 | 1.110 | 0.269 |
|  |  | Prosody | 1.11 | 1.03 | 0.671 | 0.504 |
|  |  | Demo. duration *(s)* | 4.10 | 4.25 | 0.948 | 0.345 |
|  |  | Time to Demo. *(s)* | 7.81 | 8.42 | 0.593 | 0.554 |
|  | ***Infant*** | Attention *(%)* | 0.98 | 0.98 | 0.310 | 0.757 |
|  |  | Time handling objects *(s)* | 47.29 | 44.83 | 1.017 | 0.312 |

*^1^ Description of measures:* **Attention Getters** - *number of times the experimenter attempted to attract the infant’s attention during the demonstrations (e.g. “Look!”);* **Positive Commenting** - *number of times the experimenter positively commented on the object and/or action performed (e.g. “Wow!”);* **Information** – *number of times the experimenter provided information on the object and/or action (e.g. “I can brush my hair with it!”);* **Prosody** – *experimenter’s speech, rated on a scale from 1 to 5 (1=infant-directed speech, 5=adult-directed speech);* **Demo. Duration** – *duration of the demonstrations of the target actions;* **Time to Demo.** – *time elapsed between beginning of trial and beginning of a demonstration;* **Attention** – *proportion of the demonstration that the infant visually attended to;* **Time handling objects** – *cumulative time the infant spent handling the objects during test phase (out of 60s).*
